# 25(OH)D_3_ and 1.25(OH)_2_D_3_ inhibits TNF-α expression in human monocyte derived macrophages

**DOI:** 10.1371/journal.pone.0215383

**Published:** 2019-04-12

**Authors:** Aisha Rafique, Lars Rejnmark, Lene Heickendorff, Holger Jon Møller

**Affiliations:** 1 Department of Clinical Biochemistry, Aarhus University Hospital, Aarhus, Denmark; 2 Department of Endocrinology MEA, Aarhus University Hospital, Aarhus, Denmark; National Institutes of Health, UNITED STATES

## Abstract

**Purpose:**

We wanted to investigate effects of vitamin D_3_ (25(OH)D_3_ and 1.25(OH)_2_D_3_) on inflammatory cytokine expression in both activated and non-activated Mφ.

**Materials and methods:**

Mononuclear cells, isolated from healthy donor buffy coats were cultured for a 6-day differentiation-period. Fully differentiated Mφ were pre-treated with either 25(OH)D_3_ or 1.25(OH)_2_D_3_ for (4, 12 or 24 hours) +/-LPS challenge for 4 hours_._ Gene expression analyses of VDR, Cyp27b1 and pro-inflammatory markers TNF-α, IL-6, NF-κB, MCP-1, was performed using RT-quantitative PCR. TNF-α protein levels from Mφ culture media were analysed by ELISA.

**Results:**

Both 25(OH)D_3_ and 1.25(OH)_2_D_3_ significantly inhibited TNF-α expression in both LPS-stimulated and unstimulated Mφ. Also, NF-κB, and to a lesser extend IL-6 and MCP-1 were inhibited. LPS up-regulated Cyp27b1 gene expression which was partly reverted by 1.25(OH)_2_D_3_.

**Conclusion:**

These data show anti-inflammatory effects of vitamin D_3_ (25(OH)D_3_ and 1.25(OH)_2_D_3_) in human macrophages, and support, that means for targeting high dose vitamin D_3_ to the immune system may have beneficial clinical effect in inflammatory conditions.

## Introduction

Vitamin D_3_ is a lipid-soluble steroid hormone and the active metabolite 1.25(OH)_2_D_3_ is crucial for calcium/phosphate homeostasis and bone metabolism [1]. Pre-vitamin D_3_ is produced in the skin through rapid isomerization of 7-dehydrocholesterol after exposure to sunlight and is transported by vitamin D binding protein (DBP) to the liver following conversion into 25(OH)D_3_ by 25-hydroxylase (Cyp27a1)[2]. The DBP-25(OH)D_3_ complex is taken up by megalin and cubilin in the kidney and converted by 1α-hydroxylase (Cyp27b1) into 1.25(OH)_2_D_3_ [3, 4]. Besides the classical role, 1.25(OH)_2_D_3_ has broad immunoregulatory effects on innate and adaptive immune responses. [5]. The nuclear vitamin D receptor (VDR) and Cyp27b1 are expressed in most immune cells e.g. T and B lymphocytes, monocytes, Mφ, natural killer cells and dendritic cells [6, 7]. Through interaction between VDR and 1.25(OH)_2_D_3_ and heterodimerization with retinoic X receptor (RXR), this complex binds to the Vitamin D responsive element (VDRE) in the promoter region of specific genes enabling gene transcription responsible for cell regulation and differentiation [7, 8]. Mφ are plastic, heterogenic immune cells that are able to polarise into specific phenotypes during inflammatory conditions, whether low-grade, autoimmune or infectious [9, 10]. The effects of 25(OH)D_3_ and 1.25(OH)_2_D_3_ on Mφ polarisation have been examined in cell lines e.g. human THP-1 and murine RAW 264.7, however data are not consistent and detailed knowledge about the effects of vitamin D_3_ on human Mφ is lacking, although current evidence suggests anti-inflammatory effects [11–13]. Supra-physiological concentrations of 1.25(OH)_2_D_3_ carry the risk of hypercalcemia, restricting high dose anti-inflammatory treatment. However, technologies for specific targeting of 1.25(OH)_2_D_3_ to Mφ may circumvent these obstacles [12, 13]. In this study, we have therefore investigated the anti-inflammatory effects of both physiological and supra-physiological concentrations of 25(OH)D_3_ and 1.25(OH)_2_D_3_ in Mφ.

## Materials and methods

### Purification of human mononuclear cells from buffy coats

Human buffy coats were collected anonymized during routine blood donations from volunteer donors at the Blood Bank of Aarhus University Hospital. According to Danish law, collection of buffy coats does not require separate ethical approval. 50 mL buffy coats were diluted 1:1 with 0.9% NaCl and 25 mL were carefully layered to 15 mL Histopaque-1077 (Sigma-Aldrich, Soeborg, Denmark) and centrifuged at 400 g at RT for 30 minutes. The opaque interface containing mononuclear cells was transferred to new tubes, added D-PSB/2%FCS/1mM EDTA and centrifuged at 200g for 10 minutes at RT following repeated wash/centrifuge step. Monocytes were purified by plastic adherence or CD14 positive selection. For plastic adherence 2 x 10^6^ cells/mL were incubated in T75 flasks with in RPMI 1640/PS/10% human serum (Gibco, ThermoFisher Scientific, Hvidovre, Denmark) for 1h. Non-adherent cells were removed and adherent monocytes received fresh medium containing 100 ng/mL M-CSF and 10 ng/mL GM-CSF (both from PeproTech, Stockholm, Sweden) for Mφ differentiation. For CD14 positive selection, EasySep Human CD14 Positive Enrichment kit (Cat. #18058, Stemcell Technologies, Cambridge, England) was applied. Mononuclear cell suspension was prepared at a concentration of 5x10^7^ cells/mL in D-PBS/2%FCS/1mM EDTA. EasySep protocol for CD14 positive selection was applied for the remaining purification of monocytes. Monocytes received fresh medium every second day and matured to fully differentiated Mφ after 6-days incubation period.

### Stimulation of Mφ with 25(OH)D_3_ and 1.25(OH)_2_D_3_

Differentiated M**φ** were collected, counted and tested for viability (Nucleo-Counter NC-250, ChemoMetec, Alleroed, Denmark). Two mL 1x10^^6^ MD-M**φ**s/mL were seeded per well (in six-well plates) and incubated in fresh medium (RPMI/PS/10%FCS/100ng/mL M-CSF/10 ng/mL GM-CSF) for 24 hours to adhere at 37°C in 5% CO_2_/95% air. After 24 h, culture medium was removed and plates were washed with D-PBS. M**φ** were stimulated with 25(OH)D_3_ (100 and 500 nM), 1.25(OH)_2_D^3^ (0.05 nM and 10 nM) (Cayman Chemical, Biomol, Hamburg, Germany) or 10 μM Dexamethasone in RPMI/PS/10% Charcoal Stripped FCS (CS-FCS) (Gibco, ThermoFisher Scientific) for 4, 12 and 24 hours. After wash in D-PBS, cells were stimulated with +/- LPS (1μg/mL from Escherichia coli 0111:B4 (Sigma Aldrich, Soeborg, Denmark)) for 4 hours. All culture medium was collected and M**φ** were harvested and resuspended in 350 μL buffer RLT with β-mercaptoethanol (Qiagen, Sollentuna, Sweden) and stored at -80°C for RT-PCR.

### RNA extraction and gene expression analysis by real-time quantitative PCR

Total RNA was extracted using micro- to mini-RNeasy kits (Qiagen, Sollentuna, Sweden) according to the manufacturer’s specifications and protocols. 100 ng of total RNA and in total of 40 μL reaction mixtures of 1x PCR buffer, 6.25 mM MgCL_2_, 2.5 μM Oligo(dT), 1 mM dNTP, 2.5 units/μL RT, 1 unit/μL RNase inhibitor (ThermoFisher, Hvidovre, Denmark) and ddH_2_O were synthesised to cDNA by GeneAmp PCR System 9600 thermal cycler. All reactions were performed in duplicates in a total reaction volume of 10 μL containing SYBR Green I Master mix (Roche, Amsterdam, Holland), ddH_2_O and 5 pmol/μL of each target forward and reverse primer, under the following conditions: pre-incubation at 95° for 10 min followed by cycled amplification at 95° for 10 s, annealing for 20 s, and 72° for 5 s for 50 cycles. All reactions were carried out on LightCycler 480 platform (Roche, Indiana, USA). Target genes were normalised to expression levels of stabile housekeeping gene GAPDH, calculated by Normfinder software [14]. mRNA ratios of target gene/house-keeping gene were normalized to untreated control. Table 1 contains forward/reverse primers and primer specific annealing temperatures (Table 1).

**Table 1:** Forward and reverse primers and annealing temperatures for RT-qPCR.

**Household**	**5´- sequence -3´**	**Annealing Tm**
β-Actin	GGCGGCACCACCATGTACCCT	68°
	AGGGGCGGACTCGTCATACT	
B2M	TACTCCAAAGATTCAGGTTTACTC	64°
	TTCACACGGCAGGCATAC	
GAPDH	TGATGACATCAAGAAGGTGGTGAAG	68°
	TCCTTGGAGGCCATGTGGGCCAT	
**Gen**	**5´- sequence -3´**	**Annealing Tm**
VDR	CCTCCTCCTGCTCAGATCAC	66°
	AGCCAATGACCTTTTGGATG	
Cyp27b1	GACGAAGGACCAACCAGGTA	60°
	CTTGGCCCTTCTGATCATGT	
TNF-α	TGGCGTGGAGCTGAGAGA	65°
	GCAATGATCCCAAAGTAGACCT	
IL-6	ACAGCCACTCACCTCTTC	60°
	AAGTCTCCTCATTGAATCCAG	
NF-κB	CTGGAAGCACGAATTGACAGA	62°
	TGAGGTCCATCTCCTTGGTC	
MCP-1	AGGGCTCGCTCAGCCAGATGC	68°
	ACCACTTCTGCTTGGGGTCAGC	

List of target gene forward and reverse primer along with their specific annealing temperatures applied for RT-qPCR.

### Enzyme-linked immunosorbent assay (ELISA) for TNF-α and IL-6

For measurements of TNF-α and IL-6 in Mφ culture supernatants, Human TNF-α DuoSet ELISA kits (DY210-05) and Human IL-6 DuoSet ELISA kits (DY206-05) (R&D systems Bio-techne, Abingdon, United Kingdom) were applied and manufacture’s standard protocol was followed.

### Statistical analysis

Graph Pad Prism 7 software (La Jolla, USA) was applied to prepare graphs and statistical analyses. For statistical analyses, we used a one-way ANOVA analysis and Dunnett’s multiple comparisons test to compare the means of control group with each stimulation group. All error bars are represented as standard error mean (SEM) and significance is indicated as *p = <0.05, **p = <0.01, ***p = <0.001 and ****p = < 0.0001. We also performed a repeated measures one-way ANOVA test of trend to analyse dose dependent response and all p-values are giving in the figures.

## Results

### High dose vitamin D_3_ inhibits constitutively expressed pro-inflammatory markers in unstimulated Mφ

**Fig 1:**
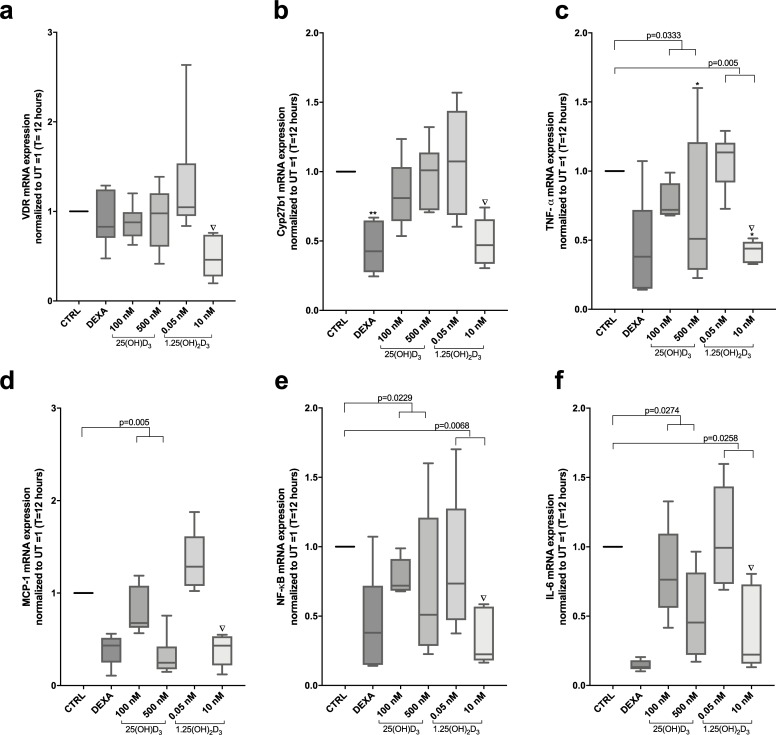
Effects of 25(OH)D_3_ and 1.25(OH)_2_D_3_ on mRNA gene expression of VDR, 1-α hydroxylase and pro-inflammatory markers in non-stimulated Mφ. Mφ (n = 6) were pre-treated with either 25(OH)D_3_ (100 nM and 500 nM) or 1.25(OH)_2_D_3_ (0.05 nM and 10 nM) for 12 hours. ∇ Symbolises an outlier, which was removed from the analysis. Following targets (**(a)** VDR, **(b)** Cyp27b1, **(c)** TNF-α, **(d)** MCP-1, **(e)** NF-κB and **(f)** IL-6 were analysed by RT-qPCR. Target mRNA gene expression was divided with reference gene expression GAPDH and the results were normalised to control Mφ given the value 1. Repeated measures One-way ANOVA Test of trend was performed to evaluate dose-dependent response of either 25(OH)D_3_ or 1.25(OH)_2_D_3_. P-value numbers are stated over the specific groups. One-way ANOVA analysis and Dunnett’s multiple comparisons test was also performed to compare the means of control group with each stimulation group and significance in illustrated as *. **(b)** ** significant difference between dexamethasone and CTRL **(c)** * Statistically significant difference between CTRL Mφ and Mφ treated with 500 nM 25(OH)D_3_.

First, we studied the effects of 25(OH)D_3_ and 1.25(OH)_2_D_3_ on VDR and Cyp27b1 mRNA expression. Four hours of treatment with 25(OH)D_3_ and 1.25(OH)_2_D_3_ did not affect VDR and Cyp27b1 gene expression (S1A and S1B Fig), but 12 hours treatment with high dose 1.25(OH)_2_D_3_ downregulated both VDR and Cyp27b1 expression (Fig 1A and 1B). A slight Cyp27b1 inhibition was observed by 25(OH)D_3_ (but not with 1.25(OH)2D_3_) after 24 hours, whereas dexamethasone inhibited Cyp27b1 gene expression at all three time points (S2B Fig). We then investigated gene expression of pro-inflammatory markers TNF-α, MCP-1, NF-κB and IL-6 in Mφ treated with 25(OH)D_3_ and 1.25(OH)_2_D_3_. The Mφ expressed low, yet detectable gene expressions of the pro-inflammatory markers, however TNF-α expression could be significantly inhibited by high dose 25(OH)D_3_ and 1.25(OH)_2_D_3_ at all time points (Fig 1C, S1 Fig and S2 Fig). The effects were comparable to the inhibitory effects of dexamethasone. Also, MCP-1 and NF-κB mRNA gene expression was considerably inhibited by high dose 25(OH)D_3_ and 1.25OH_2_D_3_ compared to untreated control Mφ at all timepoints (Fig 1D and 1E, S1 Fig and S2 Fig) comparable to the inhibitory effects of dexamethasone. High dose 25(OH)D_3_ and 1.25OH_2_D_3_ also inhibited IL-6 gene expression, however the effect was not as pronounced as seen with dexamethasone (Fig 1F, S1 Fig and S2 Fig).

### High dose vitamin D_3_ inhibits TNF-α and NF-κB in LPS stimulated Mφ

**Fig 2:**
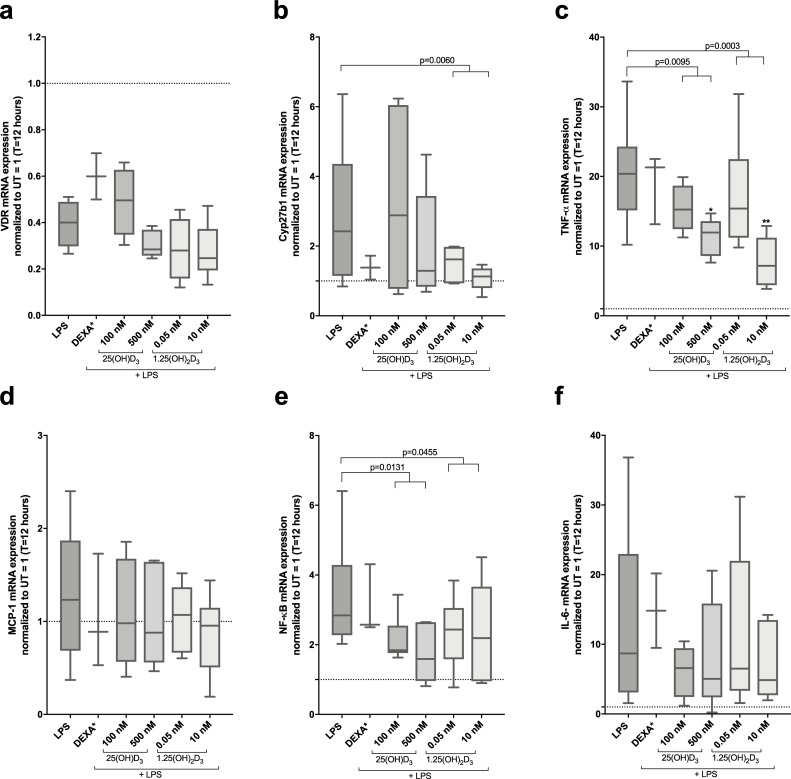
Effects of 25(OH)D_3_ and 1.25(OH)_2_D_3_ on mRNA gene expression of VDR, 1-α hydroxylase and pro-inflammatory markers LPS-induced Mφ. Mφ (n = 6) were pre-treated with either 25(OH)D_3_ (100 nM and 500 nM) or 1.25(OH)_2_D_3_ (0.05 nM and 10 nM) for 12 hours following LPS challenge (1 μg/mL) for 4 hours. Following targets (**(a)** VDR, **(b)** Cyp27b1, **(c)** TNF-α, **(d)** MCP-1, **(e)** NF-κB and **(f)** IL-6) were analysed by RT-qPCR. Target mRNA gene expression was divided with reference gene GAPDH and ratios were normalised to control Mφ given the value 1. Repeated measures One-way ANOVA Test of trend was performed to evaluate dose-dependent response of either 25(OH)D_3_ or 1.25(OH)_2_D_3_. P-value numbers are stated over the specific groups. One-way ANOVA analysis and Dunnett’s multiple comparisons test was also performed to compare the means of control group with each stimulation group and significance in illustrated as *. **(c)** * Statistically significant difference between LPS-induced Mφ and Mφ pre-treated with 500 nM 25(OH)D_3_ and ** with 10 nM 1.25(OH)_2_D_3_.

LPS strongly inhibited VDR gene expression but clearly up-regulated Cyp27b1 gene expression in Mφ similarly as reported previously [15]. Pre-treatment with 25(OH)D_3_ and 1.25(OH)_2_D_3_ did not affect VDR gene downregulation, but 1.25(OH)_2_D_3_ partly reverted Cyp27b1 gene upregulation (Fig 2A and 2B, S3 Fig and S4 Fig). As, expected, LPS strongly induced gene expressions of pro-inflammatory markers TNF-α, NF-κB and IL-6 (20, 3 and 9-fold respectively at 12 hours), whereas there was no effect on MCP-1 expression (Fig 2C, 2E and 2F).

Both 25(OH)D_3_ and 1.25(OH)_2_D_3_ significantly attenuated LPS induced TNF-α gene expression at 12 and 24 hours (Fig 2C, S3 Fig and S4 Fig). Also, attenuation of NF-κB was observed by both 25(OH)D_3_ and 1.25(OH)_2_D_3_ at 12 hours (Fig 2E), whereas no significant effect on induced MCP-1 or IL-6 expression was observed (Fig 2D–2F, S3 Fig and S4 Fig).

### High dose vitamin D_3_ inhibits TNF-α protein release in LPS stimulated Mφ

**Fig 3:**
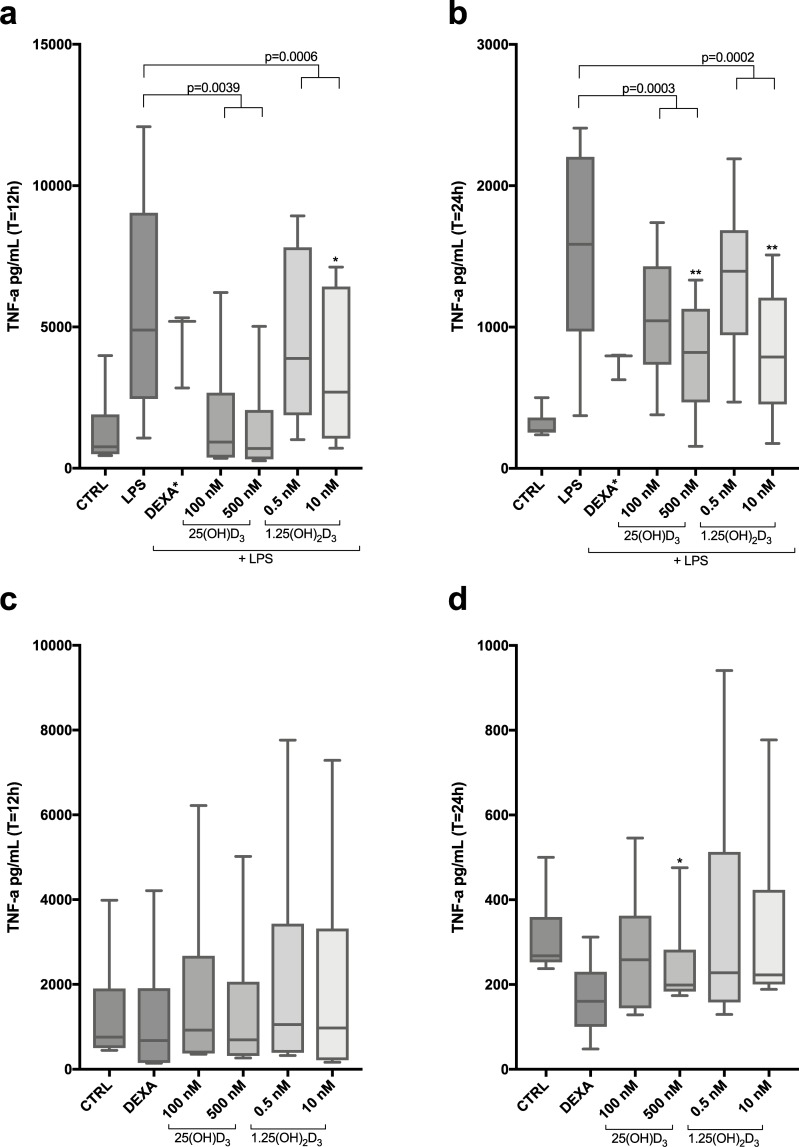
TNF-α protein secretion from LPS-induced Mφ and non-stimulated Mφ pre-treated with 25(OH)D_3_ and 1.25(OH)_2_D_3_ for T = 12 h and T = 24h. Mφ (n = 6) were pre-treated with either 25(OH)D_3_ (100 nM and 500 nM) or 1.25(OH)_2_D_3_ (0.05 nM and 10 nM) for **(a)** 12 hours followed by LPS challenge (1 μg/mL) for 4 hours or **(b)** 24 hours followed by LPS challenge (1 μg/mL) for 4 hours. **(c)** for 12 hours and **(d)** for 24 hours. TNF-α protein levels (pg/mL) were measured in Mφ culture medium by Enzyme-linked immunosorbent assay (ELISA). Repeated measures One-way ANOVA Test of trend was performed to evaluate dose-dependent response of either 25(OH)D_3_ or 1.25(OH)_2_D_3_. P-value numbers are stated over the specific groups. One-way ANOVA analysis and Dunnett’s multiple comparisons test was also performed to compare the means of control group with each stimulation group and significance in illustrated as *. **(a)** * Significant difference between LPS-induced Mφ and Mφ pre-treated with 10 nM 1.25(OH)_2_D_3_. (b) ** Significant difference between LPS-induced Mφ and Mφ pre-treated with 500 nM 25(OH)D_3_ and 10 nM 1.25(OH)_2_D_3_. **(d)** * Significant difference between control Mφ and Mφ pre-treated with 500 nM 25(OH)D_3_.

We then examined TNF-α protein secretion to culture media by ELISA. These findings confirmed the attenuation of TNF-α gene expression, showing a significant reduction in TNF-α release in LPS stimulated Mφ. In LPS stimulated Mφ, a significant reduction was seen already after 12 hours and maintained at 24 h (Fig 3A and 3B), whereas no significant change was observed in unstimulated Mφ after 12 hours, although a tendency was seen at 24 hours (Fig 3C and 3D). In addition, we also observed that high dose 25(OH)D_3_ and 1.25(OH)_2_D_3_ moderately inhibited IL-6 protein release in both un-stimulated and LPS-induced Mφ (S5 Fig).

## Discussion

The main finding of this study was to show a significant inhibition of TNF-α expression in fully differentiated human monocyte-derived Mφ by vitamin D_3_ both during normal and pro-inflammatory conditions. It has previously been shown that 1.25(OH)_2_D_3_ was able to suppress TNF-α expression in murine cell lines [16] [17] and LPS induced TNF-α gene expression in human monocytes [18]. Di Rosa et al demonstrated, that 1.25(OH)_2_D_3_ exerts diverse effects on the inflammatory response in the intermediate phases of monocyte and Mφ differentiation, including TNF-α gene suppression in TNF-α stimulated Mφ [1]. It has been suggested, that 1.25(OH)_2_D_3_ induces a switch from an “M1” Mφ phenotype, expressing iNOS, TNF-α and IL-12, to the “M2” Mφ phenotype with higher expression of CD206, Arg-1 and IL-10 and down-regulation of pro-inflammatory markers, via the VDR-PPAR-γ signalling pathway in the mouse [16]. This general shift was supported in our study by a decrease in NF-κB expression and to a lesser extend attenuated MCP-1 and IL-6 expression by both 25(OH)D_3_ and 1.25(OH)_2_D_3_. Attenuation of NF-κB by 1.25(OH)_2_D_3_ has in mice been shown to be mediated via reduced degradation of IκBα in co-transfected HEK-293 cells [19]. Suppression of MCP-1 by 1.25(OH)_2_D_3_ has previously been reported in THP-1 monocytes and PMA induced, LPS-stimulated THP-1 Mφ [20]. Interestingly, we observed similar effects of 25(OH)D_3_ and 1.25(OH)_2_D_3_ in suppression of pro-inflammatory cytokines in LPS induced Mφ. This emphasises the importance and efficiency of Cyp27b1 in the Mφ for conversion into the active metabolite. In line with this, we show a significant up-regulation of Cyp27b1 in Mφ by LPS, which was partly reverted by 1.25(OH)_2_D_3_, but not by 25(OH)D_3_. Mφ are known for their plasticity and polarisation in accordance to the surrounding microenvironment [21–23] and are known to play important roles in the development and sustaining of chronic inflammatory diseases by the production of pro-inflammatory cytokines. Of notice, TNF-α is a key mediator of inflammation evidenced by the clinical effect of TNF-α blocking biological drugs. It is therefore compelling to explore the use of high-dose vitamin D for anti-inflammatory treatment in e.g. inflammatory liver disease [24] [25] and metabolic low-grade inflammatory conditions related to insulin resistance and type 2 diabetes, where these pro-inflammatory markers are also involved [26–28]. The use of supra-physiological concentrations of 1.25(OH)_2_D_3_ as an anti-inflammatory agent, however, carries the risk of inducing hypercalcemia. To circumvent this, strategies to directly target 1.25(OH)_2_D_3_ or 25(OH)D_3_ to macrophages may be applied [25]. In summary, we have shown that 25(OH)D_3_ and 1.25(OH)_2_D_3_ supress TNF-α in fully differentiated human Mφ, both in resting/non-stimulated cells, and cells challenged by LPS. Our data support further attempts to develop systems for targeted delivery of Vitamin D to Mφ in vivo.

## Supporting information

S1 Fig Effects of 25(OH)D_3_ and 1.25(OH)_2_D_3_ treatment for 4 hours on mRNA gene expression of VDR, 1-α hydroxylase and pro-inflammatory markers in non-treated Mφ. Mφ (n = 6) were pre-treated with either 25(OH)D_3_ (100 nM and 500 nM) or 1.25(OH)_2_D_3_ (0.05 nM and 10 nM) for 4 hours. Following targets (**(a)** VDR, **(b)** Cyp27b1, **(c)** TNF-α, **(d)** MCP-1, **(e)** NF-κB and **(f)** IL-6) were analysed by RT-qPCR. Target mRNA gene expression was divided with stable reference gene GAPDH and ratios were normalised to control Mφ given the value 1. Repeated measures One-way ANOVA Test of trend was performed to evaluate dose-dependent response of either 25(OH)D_3_ or 1.25(OH)_2_D_3_. P-value numbers are stated over the specific groups. One-way ANOVA analysis and Dunnett’s multiple comparisons test was also performed to compare the means of control group with each stimulation group and significance in illustrated as *. **(c)** * significant difference between CTRL Mφ and Mφ treated with 0.05 nM 1.25(OH)_2_D_3_.
(EPS)

S2 Fig Effects of 25(OH)D_3_ and 1.25(OH)_2_D_3_ treatment for 24 hours on mRNA gene expression of VDR, 1-α hydroxylase and pro-inflammatory markers in non-treated Mφ. Mφ (n = 6) were pre-treated with either 25(OH)D_3_ (100 nM and 500 nM) or 1.25(OH)_2_D_3_ (0.05 nM and 10 nM) for 24 hours. Following targets (**(a)** VDR, **(b)** Cyp27b1, **(c)** TNF-α, **(d)** MCP-1, **(e)** NF-κB and **(f)** IL-6) were analysed by RT-qPCR. Target mRNA gene expression was divided with stable reference gene GAPDH and ratios were normalised to control Mφ given the value 1. Repeated measures One-way ANOVA Test of trend was performed to evaluate dose-dependent response of either 25(OH)D_3_ or 1.25(OH)_2_D_3_. P-value numbers are stated over the specific groups. One-way ANOVA analysis and Dunnett’s multiple comparisons test was also performed to compare the means of control group with each stimulation group and significance in illustrated as *. **(e)** * significant difference between CTRL Mφ and Mφ treated with either 100 nM or 500 nM 25(OH)D_3_ or 0.05 nM 1.25(OH)_2_D_3_.
(EPS)

S3 Fig Effects of 25(OH)D_3_ and 1.25(OH)_2_D_3_ treatment for 4 hours on mRNA gene expression of VDR, 1-α hydroxylase and pro-inflammatory markers in LPS-induced Mφ. Mφ (n = 6) were pre-treated with either 25(OH)D_3_ (100 nM and 500 nM) or 1.25(OH)_2_D_3_ (0.05 nM and 10 nM) for 4 hours following LPS challenge (1 μg/mL) for 4 hours. Following targets (**(a)** VDR, **(b)** Cyp27b1, **(c)** TNF-α, **(d)** MCP-1, **(e)** NF-κB and **(f)** IL-6) were analysed by RT-qPCR. Target mRNA gene expression was divided with reference gene GAPDH and ratios were normalised to control Mφ given the value 1. Repeated measures One-way ANOVA Test of trend was performed to evaluate dose-dependent response of either 25(OH)D_3_ or 1.25(OH)_2_D_3_. P-value numbers are stated over the specific groups. One-way ANOVA analysis and Dunnett’s multiple comparisons test was also performed to compare the means of control group with each stimulation group and significance in illustrated as *.
(EPS)

S4 Fig Effects of 25(OH)D_3_ and 1.25(OH)_2_D_3_ treatment for 24 hours on mRNA gene expression of VDR, 1-α hydroxylase and pro-inflammatory markers in LPS-induced Mφ. Mφ (n = 6) were pre-treated with either 25(OH)D_3_ (100 nM and 500 nM) or 1.25(OH)_2_D_3_ (0.05 nM and 10 nM) for 24 hours following LPS challenge (1 μg/mL) for 4 hours. Following targets were analysed by RT-qPCR (**(a)** VDR, **(b)** Cyp27b1, **(c)** TNF-α, **(d)** MCP-1, **(e)** NF-κB and **(f)** IL-6). Target mRNA gene expression was divided with reference gene GAPDH and ratios were normalised to control Mφ given the value 1. Repeated measures One-way ANOVA Test of trend was performed to evaluate dose-dependent response of either 25(OH)D_3_ or 1.25(OH)_2_D_3_. P-value numbers are stated over the specific groups. One-way ANOVA analysis and Dunnett’s multiple comparisons test was also performed to compare the means of control group with each stimulation group and significance in illustrated as *.
(EPS)

S5 Fig IL-6 protein secretion from LPS-induced Mφ and non-stimulated Mφ pre-treated with 25(OH)D_3_ and 1.25(OH)_2_D_3_ for T = 12 h. Mφ (n = 6) were pre-treated with either 25(OH)D_3_ (100 nM and 500 nM) or 1.25(OH)_2_D_3_ (0.05 nM and 10 nM) **(a)** for 12 hours **(b)** for 12 hours followed by LPS challenge (1 μg/mL) for 4 hours. IL-6 protein levels (pg/mL) were measured in Mφ culture medium by Enzyme-linked immunosorbent assay (ELISA). Repeated measures One-way ANOVA Test of trend was performed to evaluate dose-dependent response of either 25(OH)D_3_ or 1.25(OH)_2_D_3_. P-value numbers are stated over the specific groups. One-way ANOVA analysis and Dunnett’s multiple comparisons test was also performed to compare the means of control group with each stimulation group and significance in illustrated as *. **(a)** *** Significant difference between control Mφ and Mφ treated with dexamethasone.
(EPS)
